# Supplementary material for: Med12 cooperates with multiple differentiation signals to facilitate efficient lineage transitions in embryonic stem cells
Source: J Cell Sci. 2025 Apr 30;138(8):jcs263794. doi: 10.1242/jcs.263794 (PMC12079664; doi:10.1242/jcs.263794)
Supplement: Supplementary information [file joces-138-263794-s1.pdf]

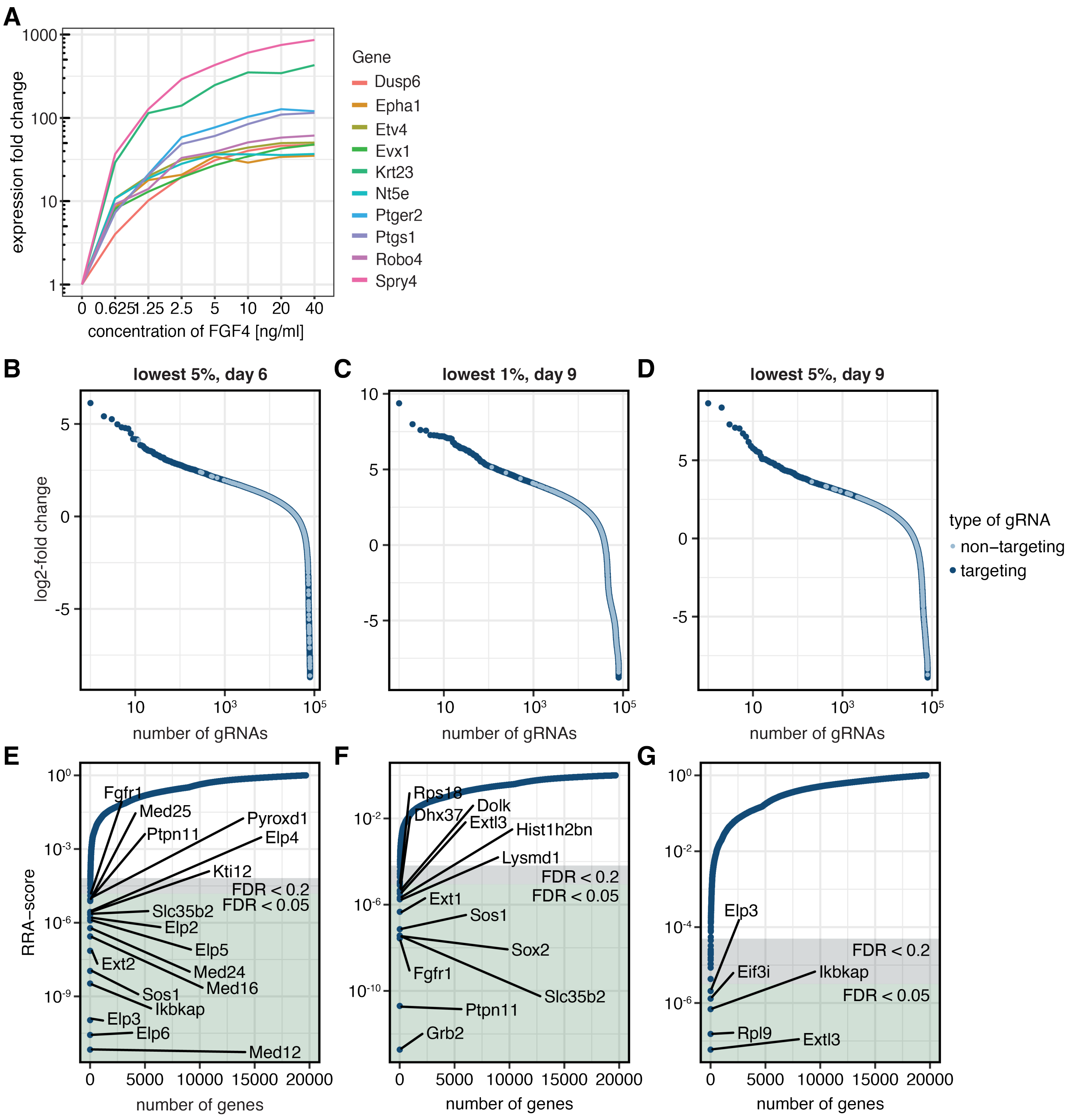

Fig. S1. Genome-wide CRISPR knockout screen based on *Spry4* as a strongly upregulated upon FGF stimulation results in robust enrichment of gRNAs and corresponding genes.

A Expression fold change of the ten most upregulated genes upon FGF4 titration in *Fgf4* mutants. *Fgf4*-mutant *Spry4*$^{H2B\text{-}Venus/+}$ cells were transitioned from 2i + LIF medium containing 10% FBS to N2B27 supplemented with Chiron and LIF for 18 h, followed by 6 h of stimulation with indicated concentrations of FGF4 in N2B27 with Chiron.

B - D Log 2-fold enrichment of gene-targeting (dark blue) and control gRNAs (light blue) in cells sorted for the lowermost 5% of H2B-Venus signal on day 6 after gRNA transduction (B), sorted for the lowermost 1% on day 9 after transduction (C), or sorted for the the lowermost 5% on day 9 after transduction (D).

E - G RRA scores for genes corresponding to enriched gRNAs identified in B - D.

Journal of Cell Science • Supplementary information

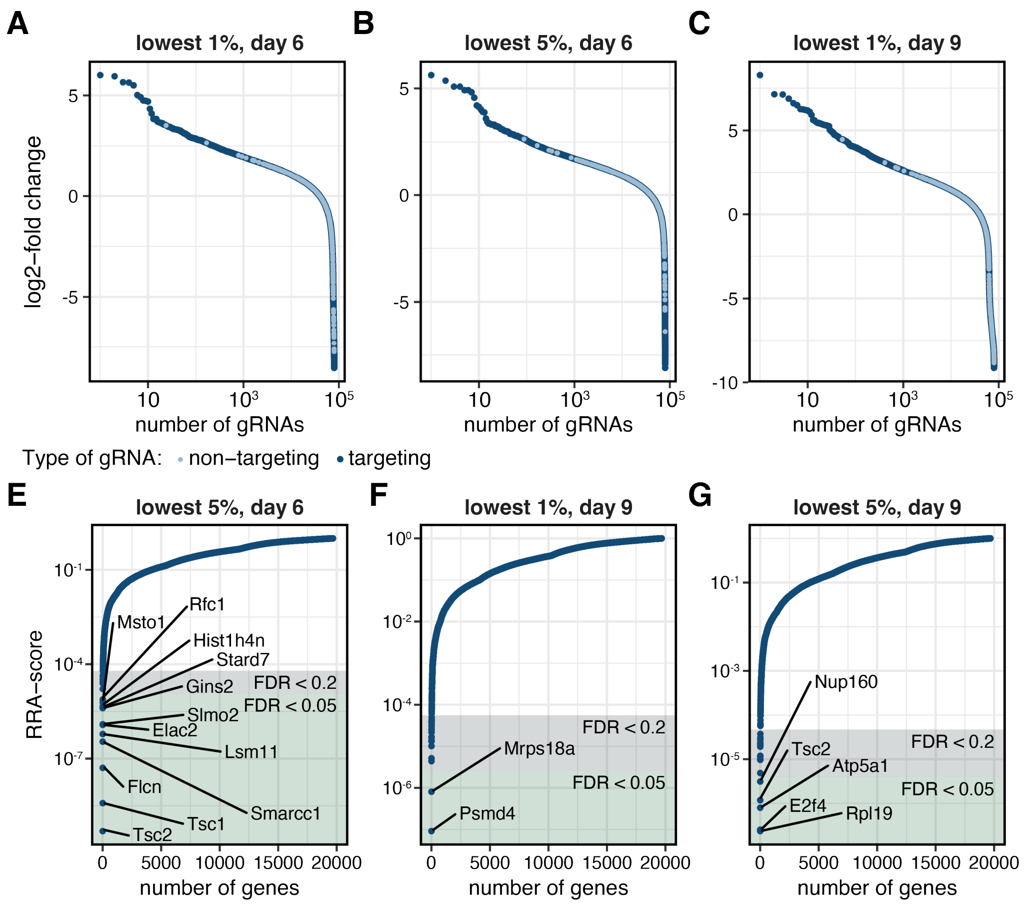

Fig. S2. Robust enrichment of gRNAs and corresponding genes that negatively regulate Spry4:H2B-Venus expression.

A - D Log 2-fold enrichment of gene-targeting (dark blue) and control gRNAs (light blue) in cells sorted for high H2B-Venus expression on different days as indicated.

E - G RRA scores for genes corresponding to enriched gRNAs identified in B - D. RRA scores for genes corresponding to gRNAs enriched in the 1% of the cells with highest fluorescence after 6 days are shown in Fig. 2A.

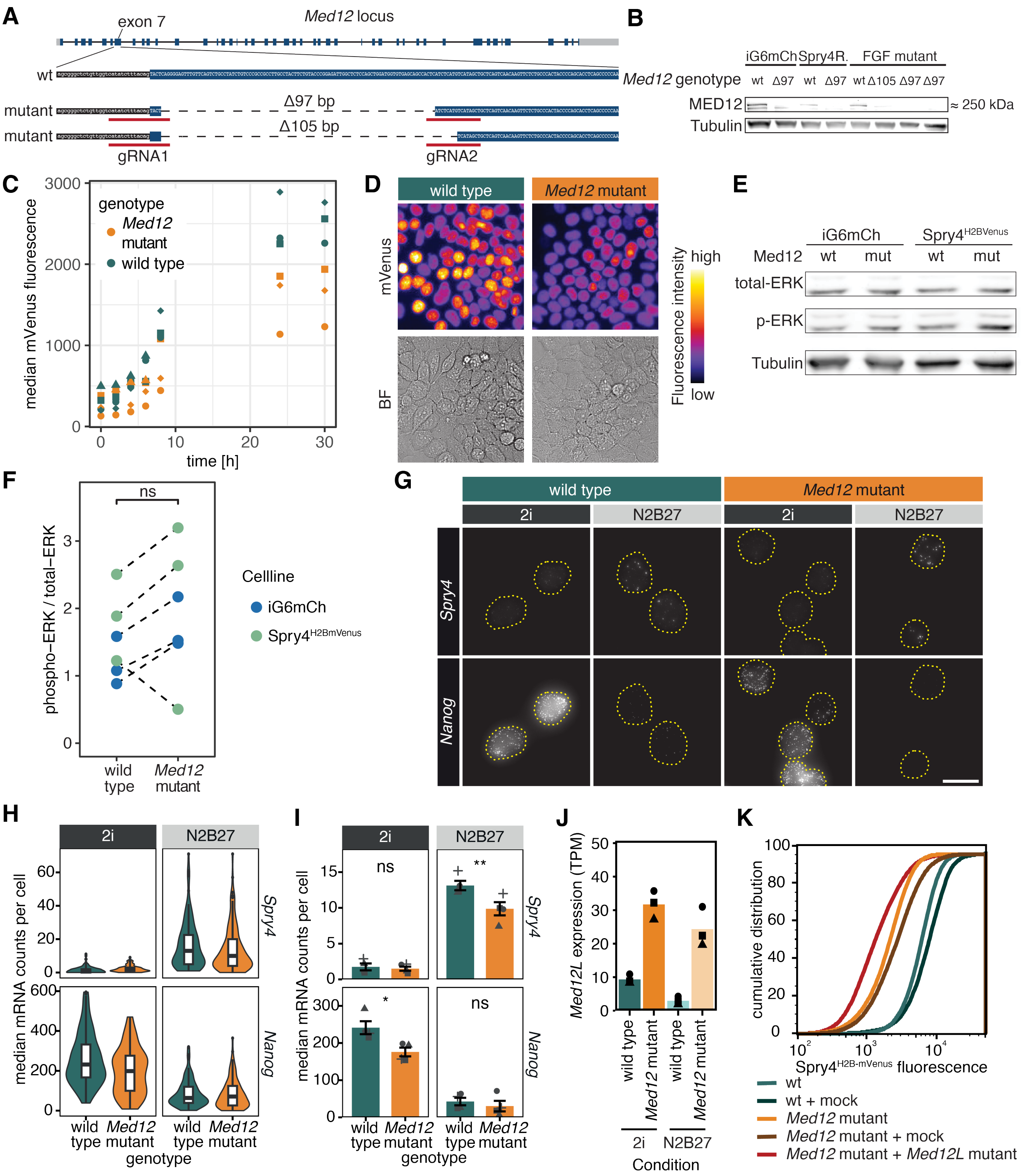

Fig. S3. Generation of *Med12* mutant cell lines.

A Schematic of the *Med12* gene locus and the gRNAs used to create a *Med12* loss-of-function by deleting 97 or 105 bp of exon7. Intronic sequence in lowercase and black underline, exonic sequence in uppercase and blue underline. **B** Immunoblotting for MED12 and Tubulin in cell lysates from independent monoclonal *Med12* mutant lines generated in different genetic genetic backgrounds. Δ97 and Δ105 indicate the type of exon7 deletion in each clone. **C** *Spry4*$^{H2B\text{-}Venus/+}$ expression upon release from 2i + LIF to N2B27 in wild-type and *Med12*-mutant cells measured by flow cytometry. Data points show median fluorescence in each experiment. N = 3.

D H2B-Venus expression in live wild-type and *Med12*-mutant *Spry4*$^{H2B\text{-}Venus/+}$ cells after 24 h of growth in N2B27 following release from 2i + LIF.

E Immunoblotting of cell lysates from *Med12* wild type and *Med12*-mutant *Spry4*$^{H2B\text{-}Venus/+}$ and iGata6 mESCs, stained for Tubulin, total- and phopsho-ERK.

F Quantification of phospho-ERK signals from immunoblots, normalized to total-ERK. N=3. ns indicates $p \geq 0.05$, paired two-sided t-test.

G Fluorescent in-situ hybridization staining of wild-type (left) and *Med12*-mutant cells (right) for *Spry4* (top) and *Nanog* (bottom) mRNAs in 2i medium, or 24h after transfer into N2B27.

H - I Violin plots (H) and summary statistics (I) of *Spry4* (top) and *Nanog* (bottom) mRNA numbers in wild-type and *Med12* mutant cells determined by in-situ hybridization as described in **G.** Violin Plots show one out of N = 4 replicates, n > 100 cells per replicate and condition.

J Expression of *Med12l* in wild-type and *Med12*-mutant cells (data from RNA-seq experiment in Fig. 3A).

K H2B-Venus expression in untransfected wild-type and *Med12*-mutant cells, and after transfection with mock or *Med12l*-targeting gRNAs. Expression was measured by flow cytometry 7 d after transfection and culture in ES + LIF medium.

Journal of Cell Science • Supplementary information

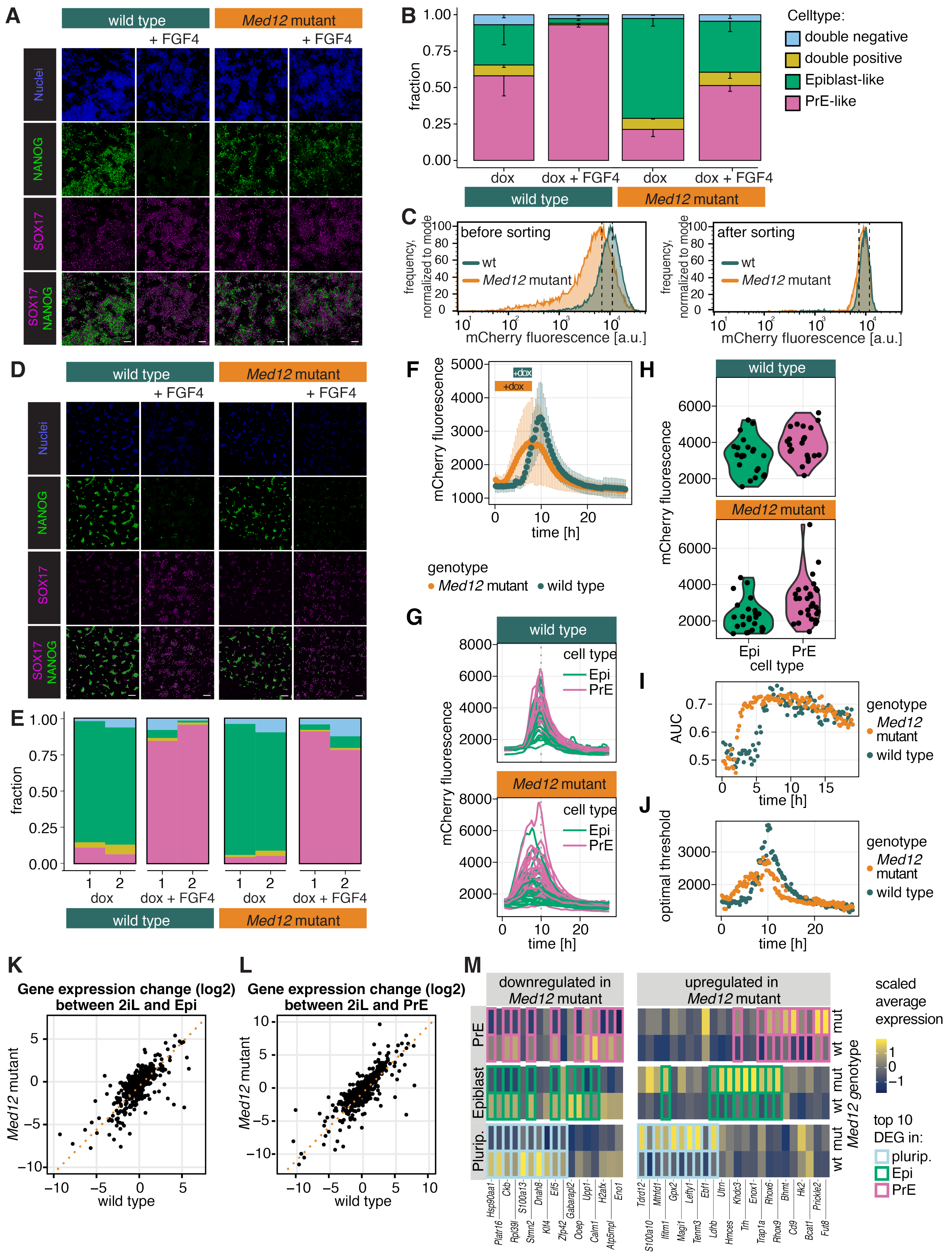

Journal of Cell Science • Supplementary information

Fig. S4 continued

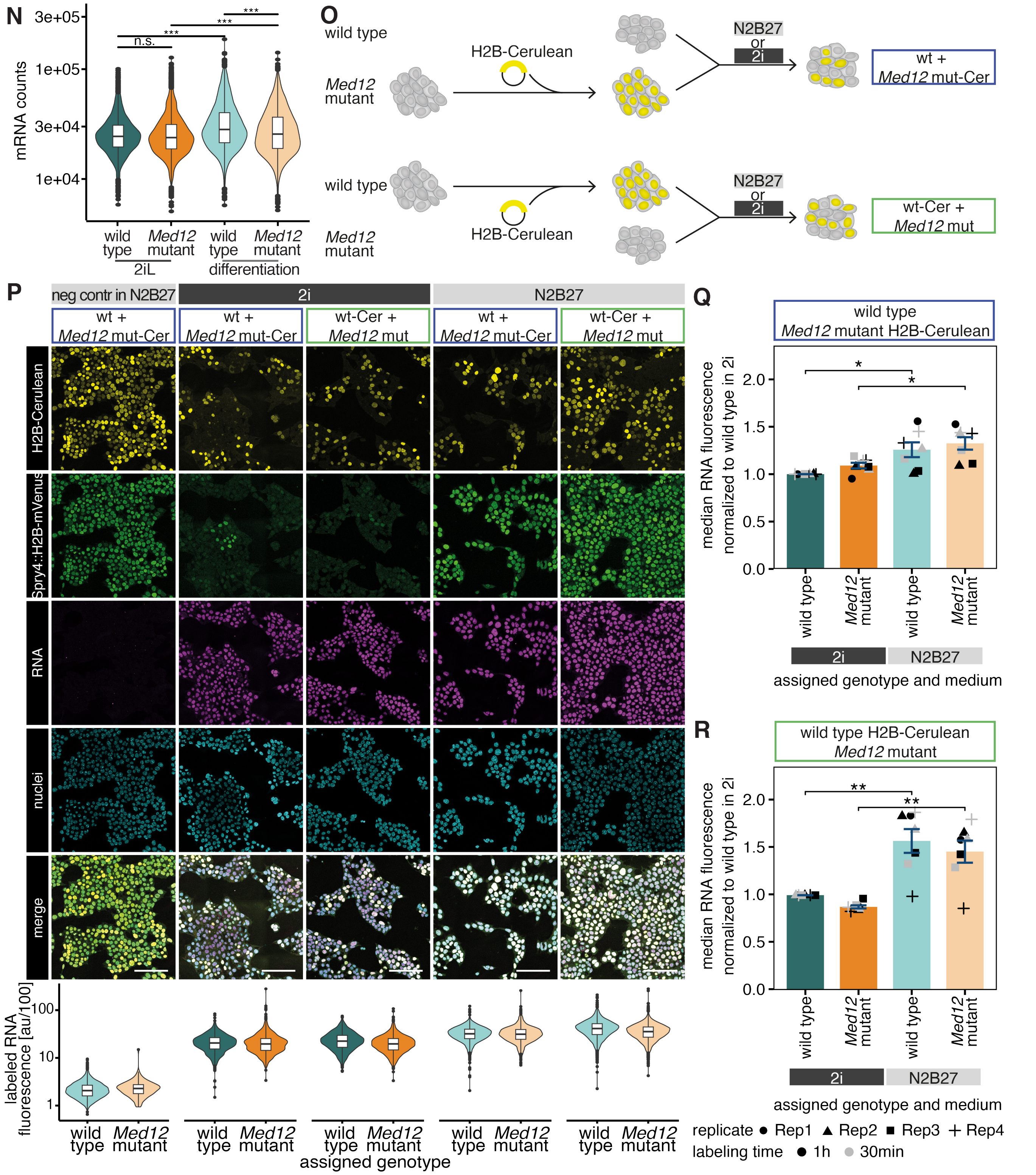

Fig. S4. PrE differentiation in *Med12* mutant cells.

A Immunostaining of the Epi-marker NANOG (green) and the PrE marker SOX17 (magenta) after 8 h of GATA6 induction and 20 h of differentiation with and without exogenous FGF4 in wild-type and *Med12*-mutant cells. Scale bar: 100 µm.

B Cell type proportions in wild-type and *Med12*-mutant cells differentiated as in (A). N=3, n > 1100 cells per replicate, error bars indicate SEM.

Journal of Cell Science • Supplementary information

C Gata6-mCherry fluorescence after 8 h of dox induction measured by flow cytometry. Left shows distribution of expression levels in the whole population, right shows expression levels after flow sorting of cells with similar fluorescence intensity. Dashed lines indicate sorting gate.

D Immunostaining of the Epi-marker NANOG (green) and the PrE marker SOX17 (magenta) after 8 h of GATA6 induction, flow sorting as described in (C), reseeding and 20 h of differentiation with and without exogenous FGF4 in wild-type and *Med12*-mutant cells. Scale bar: 100 µm.

E Cell type proportions in wild-type and *Med12*-mutant cells differentiated as in (D). N=2, n > 500 cells per replicate.

F Quantification of Gata6-mCherry expression dynamics from time-lapse movies during induction and differentiation. Boxes indicate induction times (8 h for *Med12* mutant, 4 h for wild type). Error bars indicate SD. One out of N = 5 replicates shown, n > 300 cells per time point.

G Same experiment as in (E), but showing Gata6-mCherry fluorescence in single cells. Trace color indicates differentiation outcome determined by immunostaining (Epi: green; PrE: magenta).

H Gata6-mCherry fluorescence in single cells 2 h after the end of induction, plotted separately for prospective Epi and PrE cells.

I Predictive power of GATA6-mCherry expression determined as Area Under the Curve (AUC) from ROC-analysis.

J Optimal GATA6-mCherry threshold to predict differentiation outcome determined by Youden's J statistic of ROC-analysis.

K and **L** Expression change of each gene upon differentiation from pluripotency (2iL) to Epi (K) and PrE (L) in wild-type versus *Med12*-mutant cells. Dotted, orange line indicates the unity line.

M Differentially expressed genes between *Med12* wild type and mutant cells for the three different cell states. Tile color shows scaled average gene expression, colored boxes indicate the 10 genes with the largest fold-change between *Med12* wild type and mutant cells in each cell state.

N Raw number of UMIs per cell detected by scRNAseq in wild-type and *Med12*-mutant cells. ns indicates $p \geq 0.05$, *** indicates $p \leq 0.001$, Bonferroni-adjusted Kolmogorov-Smirnov test.

O Schematic of H2B-labeling of *Med12* mutant (top) or wild type cells (bottom) to compare RNA production in pluripotency (2i) and differentiation (N2B27) conditions.

P Fluorescent images (upper panel) and violin plot of intensity (lower panel) of RNA-labeling with EU in 2i (dark grey) and N2B27 (light grey). Mixtures of genotypes were images in the same well, with one of them labeled with H2B-Cerulean (yellow) expression. For nuclei segmentation hoechst staining (cyan) was used and Spry4-H2B-mVenus-expression (green) confirmed differentiation in N2B27.

Q, R Quantification of intensity of RNA labeling based on cells assigned genotype based on their H2B-Cerulean expression (P). In (Q) the *Med12*-mutant cell line was labeled with H2B-Cerulean, in (R) the wild-type line. N = 3, n > 750 cells. n.s. indicates $p \geq 0.05$, * indicates $p < 0.05$ and ** indicates $p < 0.01$, paired two-sided t-test with Bonferroni multiple testing correction.

F - I Data from one representative experiment out of N = 3 replicates, n ≥ 45 cells per genotype.

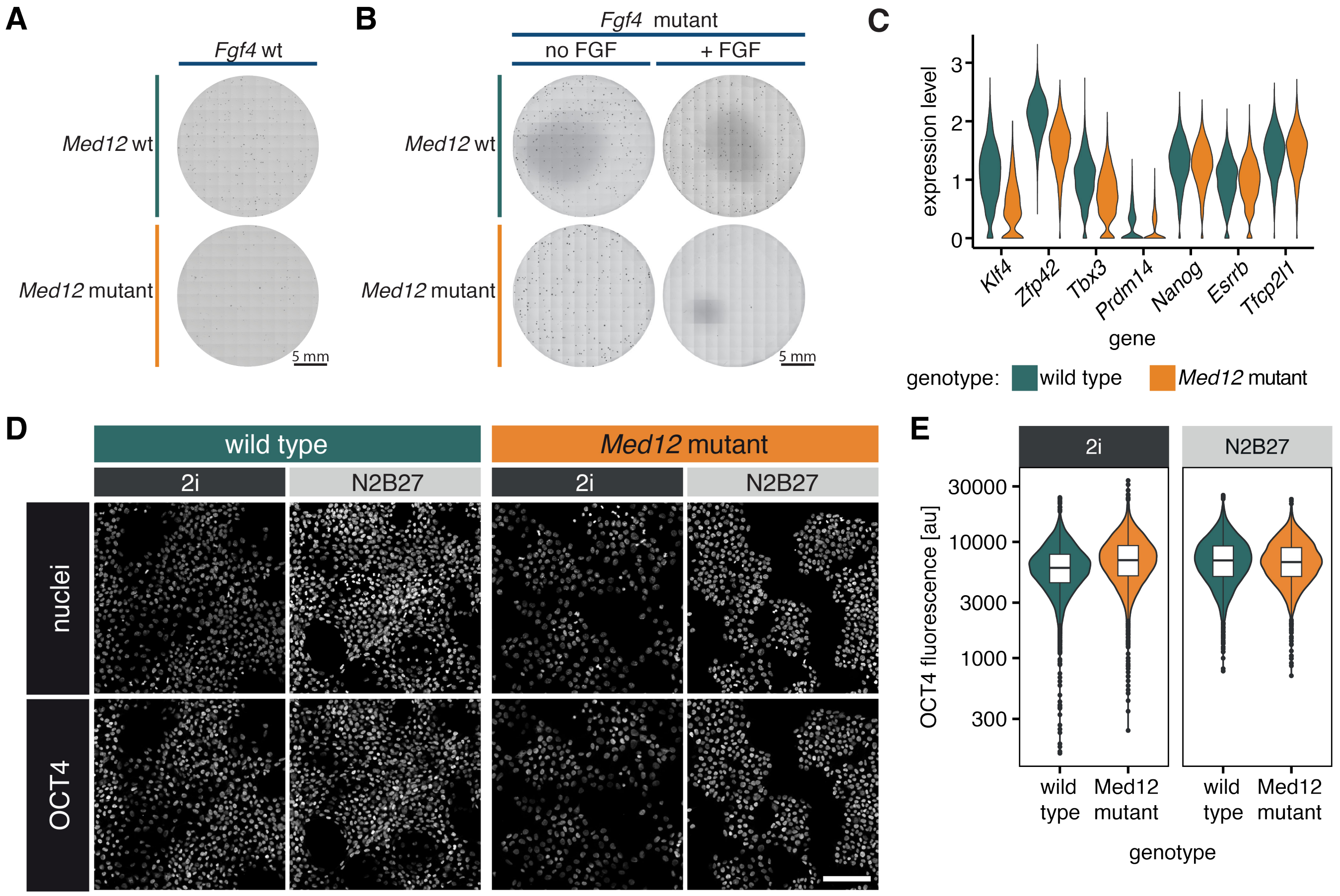

Fig. S5. Reduced clonogenicity in *Med12* mutants compared to wild type.

A and **B** Representative images of plates from the clonogenicity assay depicted in Fig. 5A, corresponding to quantifications shown in Fig. 5B. **A** shows plates with wild-type and *Med12*-mutant cells in an *Fgf4* wild type background, **B** shows plates with wild-type and *Med12*-mutant cells in an *Fgf4*-mutant background without (left) and with (right) FGF4 supplementation.

C Ln-transformed expression levels of same naïve pluripotency marker genes as in Fig. 5C,D from pluripotent cells of single cell sequencing experiment from Fig 4.

D Immunostaining for OCT4 (Pou5f1) in wild-type and *Med12*-mutant cells in 2i and N2B27. Scale bar 100 µm.

E Quantification of OCT4 fluorescence per nucleus from cells stained as in (D).

Journal of Cell Science • Supplementary information

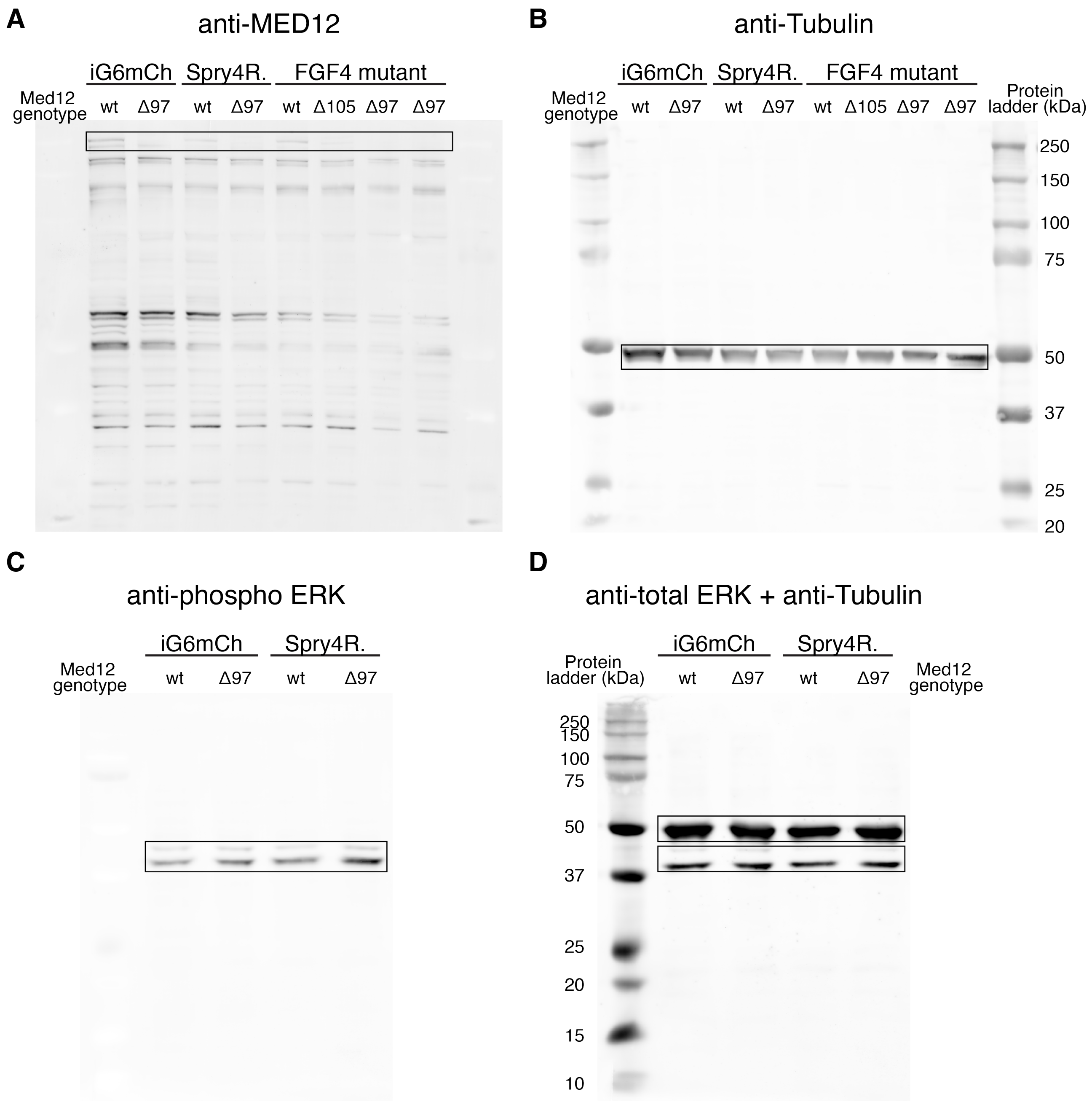

Fig. S6. Blot transparency.

A and **B** Uncropped western blot from Fig S3B showing MED12 (A) and Tubulin (B) signals in parental lines (wt) of different backgrounds (inducible GATA6-mCherry (iG6mCh), *Spry4*$^{H2B\text{-}Venus/+}$ (Spry4R.), FGF4 mutant) and *Med12*-mutant lines.

C and **D** Uncropped western blot from Fig S3E showing phospo ERK (C) and total ERK as well as Tubulin (D) signals in parental lines (wt) of different backgrounds (inducible GATA6-mCherry (iG6mCh), *Spry4*$^{H2B\text{-}Venus/+}$ (Spry4R.)) and *Med12*-mutant lines. Black rectangles indicate regions showed in FigS3.

Table S1. Raw and processed data from CRISPR screen including detected counts of gRNAs and enriched genes.

Available for download at
https://journals.biologists.com/jcs/article-lookup/doi/10.1242/jcs.263794#supplementary-data

Table S2. Differentially expressed genes comparing wild-type and *Med12*-mutant cells in 2i and after 24 h differentiation in N2B27.

Available for download at
https://journals.biologists.com/jcs/article-lookup/doi/10.1242/jcs.263794#supplementary-data

Table S3. Differentially expressed genes in *Fgf4*-mutant and *Fgf4 Med12* double mutant cells upon FGF4 stimulation in N2B27.

Available for download at
https://journals.biologists.com/jcs/article-lookup/doi/10.1242/jcs.263794#supplementary-data

Table S4. Differentially expressed genes between the Epi- (cluster 1) and PrE-cells (cluster 0) determined by single cell RNA sequencing experiment.

Available for download at
https://journals.biologists.com/jcs/article-lookup/doi/10.1242/jcs.263794#supplementary-data

Table S5. Differentially expressed genes between wild-type and *Med12*-mutant cells separately in pluripotency conditions, the Epi- and PrE-cluster.

Available for download at
https://journals.biologists.com/jcs/article-lookup/doi/10.1242/jcs.263794#supplementary-data

Table S6. Raw and normalized counts of colonies detected in the colony formation assay.

Available for download at
https://journals.biologists.com/jcs/article-lookup/doi/10.1242/jcs.263794#supplementary-data

Table S7. Oligos used as gRNAs or PCR primers.

Available for download at
https://journals.biologists.com/jcs/article-lookup/doi/10.1242/jcs.263794#supplementary-data

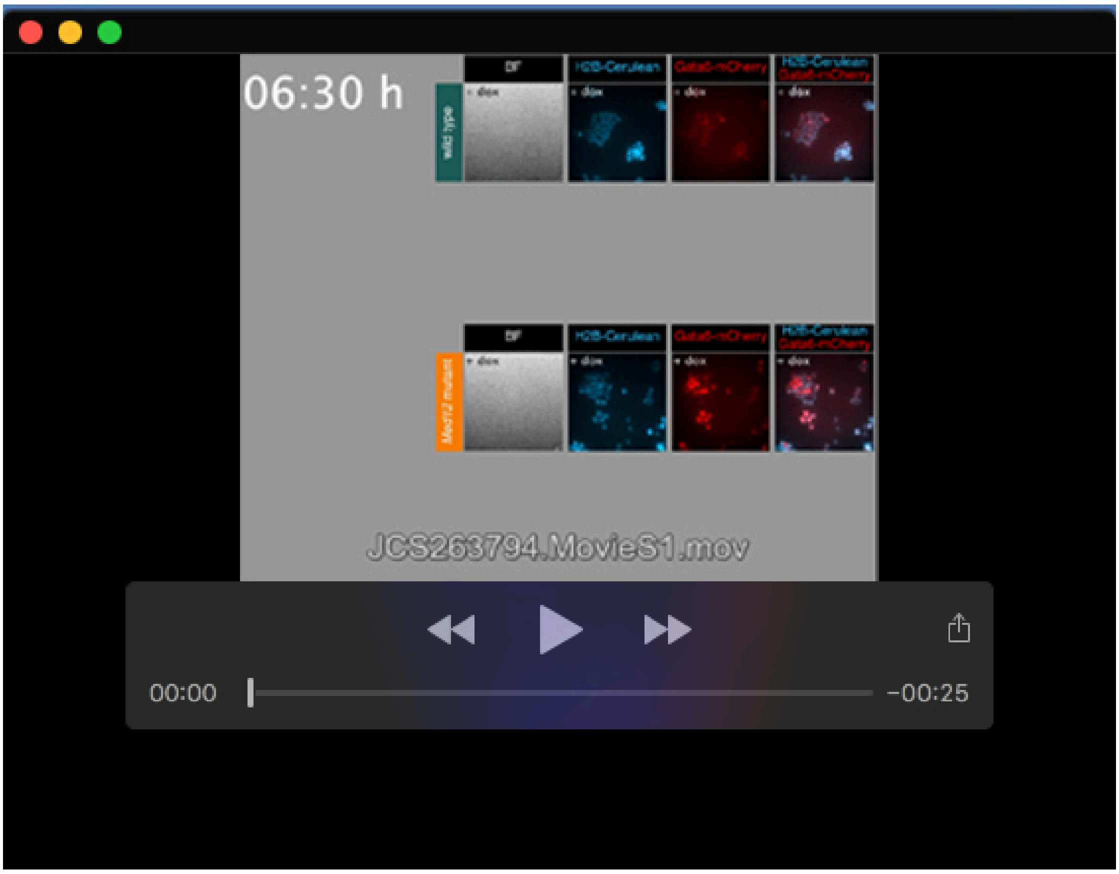

Movie 1. Timelapse imaging of H2B-Cerulean (blue) and iGata6-mCherry expression (red) in wild-type and *Med12*-mutant cells during Epi and PrE differentiation under same conditions as in Figure 3E. Following time lapse imaging, cells were immunostained for the Epi-marker NANOG (green) and the PrE marker SOX17 (magenta). Nuclei in immunostainings labelled with Hoechst dye (cyan). Scale bar: 50 µm.
