# Supplementary material for: Med12 cooperates with multiple differentiation signals to facilitate efficient lineage transitions in embryonic stem cells
Source: J Cell Sci. 2025 Apr 30;138(8):jcs263794. doi: 10.1242/jcs.263794 (PMC12079664; doi:10.1242/jcs.263794)
Supplement: Supplementary file 1 [file joces-138-263794_review_history.pdf]

Journal of Cell Science

Review COMMONS

Med12 cooperates with multiple differentiation signals to facilitate efficient lineage transitions in embryonic stem cells

Max Fernkorn and Christian Schröter

DOI: 10.1242/jcs.263794

Editor: Caroline Hill

Review timeline

Submission to Review Commons:	19 January 2024
Submission to Journal of Cell Science:	16 December 2024
Editorial decision:	26 February 2025
First revision received:	20 March 2025
Accepted:	23 March 2025

Review commons

Reviewer 1

Evidence, reproducibility and clarity

The authors describe a genome-wide CRISPR screen in mouse ES cells to identify factors and genes that regulate positively and negatively FGF/ERK signaling during differentiation. Out of known and potentially novel regulating signals, Mediator subunit Med12 was a strong hit in the screen and it was clearly and extensively shown by that the loss of Med12 results in impaired FGF/ERK signal responsiveness, modulation of mRNA levels and disturbed cell differentiation leading to reduced stem cell plasticity.

This is a very concise and well written manuscript that demonstrates for the first time the important role of Med12 in ES cells and during early cell differentiation. The results support data that had been previously observed in Med12 mouse models and in addition show that Med12 cooperates with various signaling systems to control gene expression during early lineage decision.

Fig. 3 Supp1A-B:

The loci of all three independent Med12 mutant clones and the absence of Med12 should be included. Are all three Med12 loss-of-function mutants?

Minor:

Line 466: Should be Fig. 6F, not 6E.

Significance

The CRISPR screen identified list of some novel interesting factors that regulate FGF/ERK signaling in ES cells. Med12 was then analyzed in very detail on various levels and under various differentiation conditions, resulting in a complex picture how Med12 controls stem cell plasticity. These data support results observed in mouse models and identified novel regulating mechanisms of Med12.

Reviewer 2

Evidence, reproducibility and clarity

© 2025. Published by The Company of Biologists under the terms of the Creative Commons Attribution License (https://creativecommons.org/licenses/by/4.0/).

In the manuscript "Med12 cooperates with multiple differentiation signals to enhance embryonic stem cell plasticity" Ferkorn and Schröter report on the role of Med12 in mouse embryonic stem cells. The perform an elegant genetic screen to identify regulators of Spry4 in mouse ESCs, screening for mutations that increase and decrease Spr4-reporter expression in serum/LIF conditions. They find that Med12 deletion results in defects in the exit from naïve pluripotency and in PrE-formation upon Gata-TF overexpression. Using scRNAseq experiments they report a reduction in biological noise in Med12 KO cells differentiating towards PrE upon Gata6 OE.

Major points:

1. The title might not exactly reflect the scientific findings of the manuscript. There is little direct evidence for a decrease in plasticity upon Med12 depletion.

2. Fig 1G: From the data provided it is not entirely clear how well screen results can be validated. Did some of the mutants identified in the screen also produce no detectable phenotypes? What would be the phenotype of knocking out an unrelated gene? In other words, are some of the weak phenotypes really showing Spry4 downregulation or are they withing the range of biological variance?

3. Rescue experiments by re-expressing Med12 in Med12 KO ESCs are missing. Can the differentiation and transcriptional phenotypes be rescued?

4. L365: The subheading "Transitions between embryonic... buffered against loss of Med12" is confusing. The data simply shows that Med12 KOs can still, albeit less efficiently generate PrE upon Gata TF OE. Is there evidence for some active buffering? I think the authors could simply report the data as is, stating that the phenotypes are not a complete block but an impairment of differentiation.

5. L386: Would it not make more sense to reduce dox concentrations in control cells to equalize Gata6 OE to equalize levels between Med12 KO and controls? A shorter pulse of Gata6 does not really directly address unequal expression levels due to loss of Med12. Different pulse length of OE might have consequences that the authors do not control for. This also impacts scRNAseq experiments which suffer from the same, in my opinion, suboptimal experimental setup. This is a point that needs to be addressed.

6. The reduced transcript number in Med12 KOs is interesting, but how does it come about. Is there indeed less transcriptional activity or is reduced transcript numbers a side effect of slower growth or the different cell states between WT and Med12 mutants. Appropriate experiments to address this should be performed.

7. I the proposed reduction of biological noise a feature of the PrE differentiation experiments or can it also be observed in epiblast differentiation.

8. I cannot follow the authors logic that Med12 loss results in enhanced separation between lineages. How is this experimentally supported.

Minor points:

Fig 3, Supp1 A: What exactly are the black and blue highlighted letters?

Significance

and differentiation defects were appropriately analyzed. The manuscript has some weaknesses in investigating the molecular mode of action of Med12 which could be improved to provide more significant insights.

Reviewer 3

© 2025. Published by The Company of Biologists under the terms of the Creative Commons Attribution License (https://creativecommons.org/licenses/by/4.0/).

Evidence, reproducibility and clarity

The authors sought to identify genes important for the transcriptional changes needed during mouse ES cell differentiation. They identified a number of genes and focussed on Med12, as it was the strongest hit from a cluster of Mediator components.

Using knockout ES cells, differentiation assays, bulk and scRNAseq, they clearly show that Med12 is important for transgene activation and for gene activation generally during exit from self-renewal, but it is not specifically influencing differentiation efficacy per se. Rather, cells lacking Med12 display "a reduced ability to react to changing culture conditions" and, by inference, to environmental changes. They conclude that Med12 "contributes to the maintenance of cellular plasticity during differentiation and lineage transitions."

Med12 is a structural component of the kinase module of Mediator, but it is not clear what this study tells us about Mediator function. The authors state that their results contrast with those obtained using a Cdk8 inhibitor, which resulted in increased self-renewal (lines 577-580). I'm not sure where their results show "...that loss of Med12 leads to reduced pluripotency." (lines 579-580). They do not test potency of these cells. There is reduced expression of some pluripotency-associated markers and fewer colonies formed in a plating assay, but these assays to not test cellular potency. While their phenotype certainly appears different from that reported in cells treated with Cdk8 inhibitor, it's not clear to me what to make of it, or what it might tell us about the function of the Mediator Kinase module or of Mediator. That a co-activator is important for gene expression in general, or even for gene activation upon receipt of some signal, is not really surprising.

Minor points:

It is surprising they don't relate their work to that of Hamilton et al (https://doi.org/10.1038/s41586-019-1732-z) who conclude that differentiation from the ES cell state towards primitive endoderm is compromised without Med24.

Stylistic point: please make the separation between paragraphs more obvious. With no indentation or extra spacing between paragraphs it looks like one solid mass of words.

Significance

There is a lot of careful work here, but I'm not getting a big conclusion here. Perhaps the authors could argue their main points somewhat more stridently and what we've learned beyond this current system.

Reviewer 4

Evidence, reproducibility and clarity

Fernkorn and Schröter report the results of a screen in mESCs based on modulation of the fluorescent intensity of the Spry4:H2B-Venus reporter. They identify candidate genes that both positively and negatively modulate the expression of the reporter. Amongst those, are several known regulators of the FGF pathway (transcriptional activator of Spry4) that serve as a positive control for the screen. The manuscript focuses on characterisation of Med12, and the authors conclude that Med12 does not specifically affect FGF-targets. Paradoxically, the authors show that based on the expression of key naïve markers Med12 cells show delayed differentiation. Functionally, however, Med12 mutant cells at 48hrs can form less colonies when plated back in naïve conditions (that would normally indicate accelerated differentiation). The authors conclude that Med12 mutants have "a reduced ability to react to changing culture conditions". Next, they examine the Med12 mutation affects embryonic/extraembryonic differentiation using an inducible Gata6 expression system. They show that transgene induction is slower and dampened in mutant cells and that overall the balance of fates is skewed towards embryonic cells. Finally, they use single cell RNA sequencing and observe differences in the number of mRNAs detected, as well as

© 2025. Published by The Company of Biologists under the terms of the Creative Commons Attribution License (https://creativecommons.org/licenses/by/4.0/).

the separation between clusters in the mutant cells. They conclude that the mutants have reduce transcriptional noise levels.

Overall, it was an interesting article exploring the molecular consequences of knocking out a subunit of the mediator complex. The characterisation focuses primarily on the description of the screen and the more functional consequences of the KO, rather than delving onto the molecular aspects (e.g. whether mediator complex assembly is affected, or it's binding etc). The analysis of the transcriptional noise will be of particular interest to the community, although I have some suggestions to exclude the possibility that the analysis simply reflects changes in global transcription levels. I have a small number of concerns and requests for clarification on the data but all of them should be relatively easy to address.

Major points:

- Med12, transcription levels and noise (Figure 6G, J-L). This is an intriguing observation. The labelling and multiplexing helped resolve many of the issue typically associated with comparing 10x dataset. I have two observations about this analysis:

1. Clarify how number of mRNA counts per cell is calculated (figure 6F) - the methods only described a value normalised by the total number of counts per cell. I feel this observation is key and has repercussions for the interpretation of the data (see point below) and should be independently validated (although I recognise it's difficult!). Since the authors observed differences in a randomly integrated transgene (iGata experiments), it's possible/likely that the dysregulation of transcription output is more generic. A possible suggestion is measuring global mRNA synthesis and degradation rates, either using inhibitors or by adding modified nucleotides and measuring incorporation rate and loss through pulse/chase labelling.

2. 10x is not the ideal for looking at heterogeneity/noise since it has a low capture efficiency and there are a lot of gaps/zeros in the lower expression range. Therefore, it's simply possible that mutant cells have dampened transcriptional output, meaning lowly expressed genes which in the WT contribute to the apparent heterogeneity (because there is a higher chance of not being captured), are below the 10x detection range in the mutant. This can be seen by plotting the cumulative sum of the mean gene count across each sample - the 50% mark (=mean gene count at 50% detection) reflects a measure of the "capture efficiency" (either because of technical reasons or lower mRNA input). Generally (e.g. also seen across technical repeats), the mean coefficient of variation, entropy and other measures of population heterogeneity directly scale with this "mean gene count at 50% detection", while the cell-cell correlation inversely scales with the "mean gene count at 50% detection". If this scaling relationships are observed for the WT and mutant, then it is impossible to say from the single cell RNA-seq whether the differences in heterogeneity are due to biological or technical reasons. Unfortunately, down-sampling the reads does not generally correct or normalise for this type of technical noise since the technical errors accumulate at every step of sample prep. Of course, it's possible that the technical noise in the RNAseq obfuscates real differences in the level of noise. The failure of mutant cells to re-establish the naïve network certainly suggest there is something going on. Therefore, I suggest performing the analysis of capture efficiency vs CV2 mentioned above and adjusting the discussion accordingly, and potentially perform single molecule FISH of key variable genes at the interface of the two clusters to validate the difference in heterogeneity.

- Are Oct4 levels affected? Reduction of Oct4 is sufficient to block differentiation (Radzisheuskaya et al. 2013 - PMID: 23629142).

- Med12 mutants showing transcriptionally delayed differentiation (related to figure 4C). Is this delay also reflected in the expression of formative genes? If I understand correctly, Figure 4C is made from a panel of naïve markers. It would be good to determine if the formative network is equally affected (and in the same direction - suggesting a delay), or if the transcriptional changes speak to a global dysregulation/dampened expression.

- Control for the re-plating experiments in 2i/LIF (Figure 4B). Replating in 2iLIF + FBS can have a large selective effect in certain mutant backgrounds (e.g. Nodal mutants) which don't accurately reflect the differentiation status. To exclude such effects, it would be good to repeat the replating

© 2025. Published by The Company of Biologists under the terms of the Creative Commons Attribution License (https://creativecommons.org/licenses/by/4.0/).

assays in serum-free conditions (laminin coating can help with attachment) and include undifferentiated controls to ensure that the mutant doesn't have a clonal disadvantage.

Minor points:

- I found figure 3D and the corresponding text and caption difficult to understand. It is unclear what a "footprint", "relative pathway activity" or "spearman correlation of footprint" mean. Were all the genes listed below Med12 knocked out and sequenced in this study? I suggest re-working and maybe simplifying the text and figure. In figure S1 Sup1 the authors report the dose response of targets to FGF - are those affected in the mutant?

- Similarly, it would be helpful to guide the reader through figure 5H-I and the corresponding text and caption since it's not immediately obvious how the analysis/graphs lead to the conclusion stated.

- Role of Med12 in regulating FGF signalling. There are two observations that seems a bit at odds with the text description and it would be helpful to clarify: "ppERK levels were indistinguishable between wild-type and Med12-mutant lines" (line 222) - 5/6 datapoints show an increase. "[...] overall these results argue against a strong and specific role of Med12 in regulation of FGF target genes." (line 274). If I understood correctly, ~50% of genes are differentially transcribed because of Med12 KO.

- "[...] as well as transitions between different pluripotent states" (line 41) - references missing .

- Line 447: "differentiation conditions" - it's unclear what it's mean by differentiation and how it relates to the diagram in figure 6A. Are those the 20hr cells? Do the -8h, -4hr and 0hr cells (if I understand the meaning of the diagram) cluster all together?

- The difference in dynamics of mCherry activation as a consequence of Med12 KO are not apparent from figure 5E. It might be easier to visualise this observation if x-axis was normalised to the starting point plotting "time from start of induction".

- Figure 3H/I - what does "gene expression changes" and "fold change ratio" mean?

- Line 579-580 - please clarify what is meant by "reduced pluripotency".

- Title: "enhance ESC plasticity". not sure enhance is the right word? There is no evidence that the plasticity of cells is affected.

Significance

Overall, it was an interesting article exploring the molecular consequences of knocking out a subunit of the mediator complex. The characterisation focuses primarily on the description of the screen and the more functional consequences of the KO, rather than delving onto the molecular aspects (e.g. whether mediator complex assembly is affected, or it's binding etc). The analysis of the transcriptional noise will be of particular interest to the community, although I have some suggestions to exclude the possibility that the analysis simply reflects changes in global transcription levels. I have a small number of concerns and requests for clarification on the data but all of them should be relatively easy to address.

Original submission

First decision letter

MS ID#: jcs.263794

© 2025. Published by The Company of Biologists under the terms of the Creative Commons Attribution License (https://creativecommons.org/licenses/by/4.0/).

MS TITLE: Med12 cooperates with multiple differentiation signals to facilitate efficient lineage transitions in embryonic stem cells

AUTHORS: Max Fernkorn; Christian Schröter
ARTICLE TYPE: Review Commons Transfer

We have now reached a decision on the above manuscript. I am sorry for the long wait.

To see the reviewers' reports and a copy of this decision letter, please go to:

As you will see, the reviewers gave favourable reports but there are a few small points raised by reviewer 2 that I would like you to address in the text. I hope that you will be able to carry these out quickly because I would like to be able to accept your paper.

Please ensure that you clearly highlight all changes made in the revised manuscript. Please avoid using 'Tracked changes' in Word files as these are lost in PDF conversion.

I should be grateful if you would also provide a point-by-point response detailing how you have dealt with the points raised by the reviewers in the 'Response to Reviewers' box. Please attend to all of the reviewers' comments. If you do not agree with any of their criticisms or suggestions please explain clearly why this is so.

Journal of Cell Science is committed to maintaining the integrity of the scientific record. Please read our requirements for preparing your figures to avoid a potential delay in the publication process or rejection on the basis of non-compliance with these guidelines. This guide also includes recommendations on improving figure layout to help reviewers and readers appreciate your data.

First revision

Author response to reviewers' comments

Fernkorn & Schröter, "Med12 cooperates with multiple differentiation signals to facilitate efficient lineage transitions in embryonic stem cells", response to reviewers.

We thank reviewers #1 and #3 for the positive appreciation of our work and the revisions that we have made in response to the first round of review. Both reviewers did not raise any further points to address. Reviewer #2 only suggested a small number of textual changes, that we have all attended to as outlined below.

Comments from the Reviewers:

Reviewer 2, SUGGESTIONS TO AUTHORS

1. Line 322: incomplete sentence.

This error has been fixed.

2. Line 418: Fair enough to mention enhancer-promoter interactions as this is a proposed function for Mediator. However after mentioning this, the authors do not address it directly. I.e. if they are going to bring this up here, then I expect to see some experiments directly addressing this, and these are not here. I would remove this from this part, and in the Discussion (lines 499-500) it can be brought up, but I'd expect them to explain more thoroughly why they think this is relevant to their findings.

We thank the reviewer for pointing out that mentioning a potential role of Med12 in establishing enhancer-promoter interactions in the results section is potentially distracting. We have therefore

© 2025. Published by The Company of Biologists under the terms of the Creative Commons Attribution License (https://creativecommons.org/licenses/by/4.0/).

removed this aspect from the opening paragraph of the results section related to Fig. 5 (line 327). Instead, we have brought together some of the speculation how this function of Med12 could explain our results to the discussion section (lines 448, 449), to explain this idea more thoroughly.

3. Lines 435-436: "...these results suggest that loss of Med12 impairs the ability of single cells to re-establish a gene expression profile compatible with growth in 2i + LIF..."

I don't see that they suggest this is about re-establishment. It could equally be about time it takes to exit the naïve state. In fact the latter seems to be more likely the case, based on the following experiments.

To address the reviewer's concern, we have reworded this sentence and simply state that "loss of *Med12* impairs the ability of single cells to form naïve pluripotent colonies in 2i + LIF medium following transient differentiation in N2B27." (Lines 350 and 351).

4. Lines 526-527: "These opposing phenotypes indicate that MED12 has functions that are independent from the CDK8-module" I disagree, since we don't know what MED12 is doing within the kinase module. It could be acting to mitigate the function of this module, for example.

This is a good point. We now write that "[These opposing phenotypes]... argue against a positive role of *Med12* for the activity of the CDK8-module" (lines 427, 428), and have relegated the speculation that Med12 might have CDK8-independent functions to the end of that paragraph (lines 433, 434).

5. Lines 533-534: "Med12 has been found to maintain pluripotency not only in the epiblast, but also in the trophoectoderm". Trophectoderm is not pluripotent.

Although the title of the cited paper explicitly mentions the term "trophoblast pluripotency", we agree with the reviewer and have changed this sentence to "*Med12* has been found not only to preserve pluripotency in the epiblast, but also to maintain an undifferentiated state of the trophoblast".

<u>Second decision letter</u>

MS ID#: jcs.263794R1

MS TITLE: Med12 cooperates with multiple differentiation signals to facilitate efficient lineage transitions in embryonic stem cells

AUTHORS: Max Fernkorn; Christian Schröter
ARTICLE TYPE: Review Commons Transfer

I am happy to tell you that your manuscript has been accepted for publication in Journal of Cell Science, pending standard publication integrity checks.

Your manuscript is now with our production department, and if we require anything further from you in terms of source files, we will be in touch shortly. Otherwise, you will receive proofs in due course.

To see the reviewers' reports and a copy of this decision letter, please go to:

You may be eligible for free or discounted Open Access publication as part of our Read & Publish initiative. More details of this can be found below. If you haven't already selected Open Access and would like to, please contact our editorial office (jcs@biologists.com) within 24 hours of this email. Authors who have selected Open Access will receive a transaction link by separate email.

© 2025. Published by The Company of Biologists under the terms of the Creative Commons Attribution License (https://creativecommons.org/licenses/by/4.0/).
